# *Pterodon emarginatus* oleoresin-based nanoemulsion as a promising tool for *Culex quinquefasciatus* (Diptera: Culicidae) control

**DOI:** 10.1186/s12951-016-0234-5

**Published:** 2017-01-03

**Authors:** Anna E. M. F. M. Oliveira, Jonatas L. Duarte, Rodrigo A. S. Cruz, Raimundo N. P. Souto, Ricardo M. A. Ferreira, Taires Peniche, Edemilson C. da Conceição, Leandra A. R. de Oliveira, Silvia M. M. Faustino, Alexandro C. Florentino, José C. T. Carvalho, Caio P. Fernandes

**Affiliations:** 1 Laboratório de Pesquisa em Fármacos, Curso de Farmácia, Universidade Federal do Amapá, Campus Universitário Marco Zero do Equador, Rodovia Juscelino Kubitschek de Oliveira, KM-02 Bairro Zerão, Macapá, AP CEP 68902-280 Brazil; 2 Laboratório de Nanobiotecnologia Fitofarmacêutica, Curso de Farmácia, Universidade Federal do Amapá, Campus Universitário Marco Zero do Equador, Rodovia Juscelino Kubitschek de Oliveira, KM-02 Bairro Zerão, Macapá, AP CEP 68902-280 Brazil; 3 Laboratório de Artrópodes, Universidade Federal do Amapá, Curso de Ciências Biológicas, Universidade Federal do Amapá, Campus Universitário Marco Zero do Equador, Rodovia Juscelino Kubitschek de Oliveira, KM-02 Bairro Zerão, Macapá, AP CEP 68902-280 Brazil; 4 Laboratório de Pesquisa, Desenvolvimento e Inovação em Bioprodutos, Universidade Federal de Goiás, Faculdade de Farmácia, Praça Universitária, 1166, Setor Leste Universitário Universitário, Goiânia, GO CEP 74605220 Brazil; 5 Laboratório de Cultivo de Algas, Curso de Farmácia, Universidade Federal do Amapá, Campus Universitário Marco Zero do Equador, Rodovia Juscelino Kubitschek de Oliveira, KM-02 Bairro Zerão, Macapá, AP CEP 68902-280 Brazil; 6 Laboratorio de Absorção Atomica e Bioprospecção, Curso de Farmácia, Universidade Federal do Amapá, Campus Universitário Marco Zero do Equador, Rodovia Juscelino Kubitschek de Oliveira, KM-02 Bairro Zerão, Macapá, AP CEP 68902-280 Brazil

**Keywords:** Larvicidal, Nanoemulsion, Oleoresin, Sucupira

## Abstract

**Background:**

Preparation of nanoformulations using natural products as bioactive substances is considered very promising for innovative larvicidal agents. On this context, oil in water nanoemulsions develop a main role, since they satisfactorily disperse poor-water soluble substances, such as herbal oils, in aqueous media. *Pterodon emarginatus*, popularly known as sucupira, has a promising bioactive oleoresin. However, to our knowledge, no previous studies were carried out to evaluate its potential against *Culex quinquefasciatus*, the main vector of the tropical neglected disease called lymphatic filariasis or elephantiasis. Thus, we aimed to investigate influence of different pairs of surfactants in nanoemulsion formation and investigate if a sucupira oleoresin-based nanoemulsion has promising larvicidal activity against this *C. quinquefasciatus*. We also evaluated morphological alteration, possible mechanism of insecticidal action and ecotoxicity of the nanoemulsion against a non-target organism.

**Results:**

Among the different pairs of surfactants that were tested, nanoemulsions obtained with polysorbate 80/sorbitan monooleate and polysorbate 80/sorbitan trioleate presented smallest mean droplet size just afterwards preparation, respectively 151.0 ± 2.252 and 160.7 ± 1.493 nm. They presented high negative zeta potential values, low polydispersity index (<0.300) and did not present great alteration in mean droplet size and polydispersity index after 1 day of preparation. Overall, nanoemulsion prepared with polysorbate 80/sorbitan monooleate was considered more stable and was chosen for biological assays. It presented low LC_50_ value against larvae (34.75; 7.31–51.86 mg/L) after 48 h of treatment and some morphological alteration was observed. The nanoemulsion did not inhibit acetylcholinesterase of *C. quinquefasciatus* larvae. It was not toxic to green algae *Chlorella vulgaris* at low concentration (25 mg/L).

**Conclusions:**

Our results suggest that optimal nanoemulsions may be prepared with different surfactants using a low cost and low energy simple method. Moreover, this prototype proved to be effective against *C. quinquefasciatus*, being considered an ecofriendly novel nanoproduct that can be useful in integrated control programs of vector control.

## Background

*Culex quinquefasciatus* (Diptera: Culicidae) is a nocturnal domestic mosquito with high occurrence on tropical and subtropical regions [1]. Often, its population density is associated to deforestation and urbanization process [2–4]. *C.* quinquefasciatus deposits its eggs and develops on standing water with high concentration of organic material. Thus, it is associated to substandard housing, absence of basic sanitation, treated water and others [5, 6]. Moreover, hematophagic-feeding habits favors *C. quinquefasciatus* proliferation close to human population [7]. This species is highly anthropophilic and responsible by transmission of filarial nematodes, which cause several diseases in humans [8].

Lymphatic filariasis is caused by the nematode parasites *Wuchereria bancrofti*, *Brugia malayi* and *Brugia timori* [9]. In Brazil, etiological agent of this disease is *Wuchereria bancrofti* [2]. The cycle of the disease begins during blood repast, when the infected female of the vector transmit *W. bancrofti* larvae to human host. These larvae migrate to lymphatic system and develop to adult stage, causing dilatation of vessels [1, 10]. This disease is also known as elephantiasis and it is recognized as a neglected disease associated to poverty [11, 12] with high prevalence in tropical and sub-tropical countries [13]. According to World Health Organization (WHO), it is estimated that around 120 million of people worldwide has lymphatic filariasis [14]. Various form of manifestation of this disease include asymptomatic behavior and physical incapacity [15], mainly due to chronical hydrocele and elephantiasis of the legs or arms [16]. More than 40 million of people around the world were marginalized until 2012 [14]. On this context, WHO launched a global program for eradication of lymphatic filariasis until the year 2020 [9]. The main strategy involves treatment of population on endemic areas, control of morbidity and prevent incapacity that is associated to the disease [14]. However, another potential alternative involves environmental control, aiming to interrupt transmission by the vector.

Growing interest is observed worldwide for new integrative practices for vector control. Several of them involve utilization of natural products as bioactive agents. These compounds are biodegradable and may be used as potent ecofriendly insecticides [17]. A new approach relies on utilization of these plant-derived insecticides to prepare nanosize products [18]. The nano-scale allows achievement of optimized properties regarding biological activities, chemical and physical stability, making them versatile innovative products [19]. On this context, several nanoformulations can be prepared, including nanoemulsions. They are dispersed systems with sub-micrometer size droplets, often ranging from 20 to upper limits between 100 and 500, according to different author criteria [20]. Nanoemulsions have been considered very promising to enhance solubility of poor water-soluble substances [21]. On this context, development of herbal bioactive oil-based nanoemulsions has great potential for mosquito larvae control [22–25]. Moreover, several effective nanoemulsions containing natural oils were considered effective larvicidal agents against *C. quinquefasciatus* [26–28].

*Pterodon emarginatus* Vogel is a traditional plant species with a wide range of folk use in Brazil, being popularly known as “sucupira” or “sucupira-branca” [29]. Terpenoids from seeds of sucupira, especially vouacapan diterpenes, develop a main role as bioactive compounds of this plant [30, 31], being major constituents of the ambar coloured oleoresin obtained from its seeds. This oily material was subjected to some studies aiming to develop emulsions with submicrometer droplets [29, 32]. Considering the larvicidal potential of sucupira oleoresin and its terpenoids against *Aedes aegypti* [33, 34], a promising larvicidal nanoemulsion against this vector larvae was prepared using this raw material [25]. However, to our knowledge, no studies were carried out for another pest and/or vector insects. Thus, as part of our ongoing studies with larvicidal natural product-based nanoemulsions, the present study aim to evaluate insecticidal activity of sucupira-based ecofriendly nanoemulsion against *Culex quinquefasciatus*.

## Results and discussion

Table 1 shows droplet size, particle size distribution (polydispersity index) and zeta potential of formulations prepared with sucupira oleoresin. All of them presented high negative zeta potential values. Adsorption of hydroxyl groups and/or conjugated bases of secondary metabolites, which naturally occur on some natural oils, at the surface of micelles has been associated to this phenomenon [35]. Thus, dissociation of some substances from sucupira oleoresin, such as fatty acids and others (e.g. vouacapan diterpene acids) may be responsible by this observation. Most of them presented high mean droplet size (>200 nm) and high polydispersity index (>0.500), in addition to large amount of precipitate. Nanoemulsions obtained with polysorbate 80/sorbitan monooleate and polysorbate 80/sorbitan trioleate presented smallest mean droplet size just afterwards preparation, respectively 151.0 ± 2.252 and 160.7 ± 1.493 nm. They also presented fine appearance, translucent aspect and bluish reflect, which are in accordance with the concept of nanoemulsions [20]. Influence of surfactant type, as well as required hydrophile-lypophile balance (rHLB) value of the oil, is a major factor of influence on nanoemulsion formation. Regarding literature data, it can be observed that utilization of different pairs of sorbitan alkanoates/ethoxylated sorbitan alkanoates at rHLB of different oils (interval between 11 and 12) also successfully generated nanoemulsions with mean droplet size below 200 nm [36]. Thus, considering surfactant pairs employed in the present study, our results suggest that polysorbate 80/sorbitan monooleate and polysorbate 80/sorbitan trioleate may be considered the best pairs for preparation of sucupira oil based nanoemulsions.

**Table 1:** Droplet size, polydispersity index and zeta potential of nanoemulsions prepared with sucupira oleoresin and different pairs of surfactants (rHLB = 11)

		Size (nm)	PDI	Zeta potential (mV)	Size (nm)	PDI	Zeta potential (mV)
		Day 0			Day 1		
T80	S80	151.0 ± 2.252	0.221 ± 0.006	−32.5 ± 1.07	146.3 ± 1.450	0.219 ± 0.005	−30.5 ± 0.819
T20	S80	2540.0 ± 996.7	1.000 ± 0.000	−52.6 ± 4.14	1461.0 ± 470.8	1.000 ± 0.000	−43.8 ± 0.603
T80	TS	160.7 ± 1.493	0.252 ± 0.012	−29.2 ± 0.346	159.8 ± 3.46	0.277 ± 0.027	−31.4 ± 0.404
T20	TS	650.0 ± 663.9	0.756 ± 0.200	−31.3 ± 1.08	526.2 ± 397.2	0.601 ± 0.194	−34.3 ± 1.11
MP400		223.7 ± 28.69	0.391 ± 0.034	−43.2 ± 0.82	352.3 ± 198.8	0.536 ± 0.078	−40.6 ± 1.10
MP600	DP600	507.1 ± 171.6	0.576 ± 0.109	−35.8 ± 1.00	345.1 ± 161.1	0.568 ± 0.025	−30.3 ± 0.34
MP600	DP400	464.9 ± 176.1	0.569 ± 0.085	−44.0 ± 0.351	345.7 ± 83.2	0.476 ± 0.078	−39.3 ± 0.569
T20	DP600	750.8 ± 238.2	0.763 ± 0.063	−40.7 ± 0.451	322.6 ± 49.73	0.579 ± 0.092	−35.5 ± 0.200
T20	DP400	460.2 ± 134.6	0.608 ± 0.125	−52.2 ± 1.38	484.1 ± 59.68	0.576 ± 0.103	−51.6 ± 1.71
T80	DP600	1055 ± 65.11	0.779 ± 0.026	−14.3 ± 0.289	898.2 ± 141.1	0.921 ± 0.136	−37.3 ± 0.208
T80	DP400	248.3 ± 30.51	0.605 ± 0.157	−49.7 ± 1.16	214.5 ± 18.6	0.504 ± 0.060	−44.0 ± 3.67

Results are expressed as mean ± standard deviation

T80 = polysorbate 80. S80 = sorbitan monooleate. T20 = polysorbate 20. TS = sorbitan trioleate. DP400 = polyethyleneglycol 400 dioleate. DP600 = polyethyleneglycol 600 dioleate. MP400 = polyethyleneglycol 400 monooleate. MP600 = polyethyleneglycol 600 monooleate

**Fig. 1:**
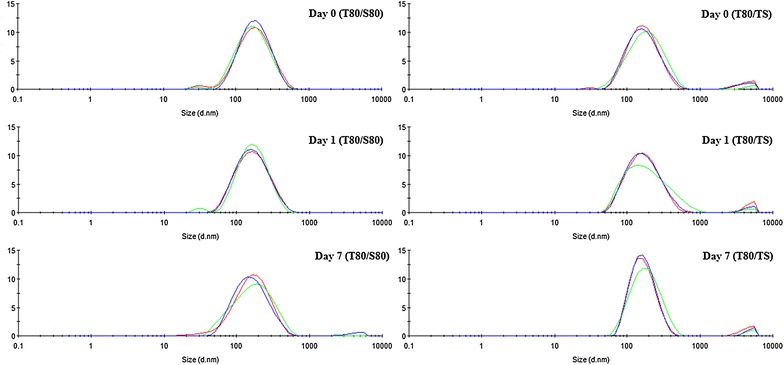
Particle size distribution of *P. emarginatus* nanoemulsions prepared with different surfactant pairs. Mean droplet size S80/T80. Day 0: 151.0 ± 2.252 nm; Day 1: 146.3 ± 1.450 nm; Day 7: 141.6 ± 0.9504 nm. Polydispersity index. Day 0: 0.221 ± 0.006; Day 1: 0.219 ± 0.005; Day 7: 0.245 ± 0.004. Mean droplet size T80/TS. Day 0: 160.7 ± 1.493 nm; Day 1: 159.8 ± 3.460 nm; Day 7: 167.9 ± 1.473 nm. Polydispersity index. Day 0: 0.252 ± 0.012; Day 1: 0.277 ± 0.027; Day 7: 0.231 ± 0.012

Sucupira oil based nanoemulsions prepared with polysorbate 80/sorbitan monooleate and polysorbate 80/sorbitan trioleate did not present great alteration in mean droplet size and polydispersity index after 1 day of preparation (Table 1). Moreover, we observed high homogeneity of particle size and almost monomodal distribution even after 7 days of storage (Fig. 1). Regarding physical stability of these nanoemulsions, we observed that the one prepared with polysorbate 80/sorbitan trioleate presented slight few precipitate after 7 days. Sucupira oil has several bioactive substances with low water solubility, such as diterpenes. These terpenoid substances are often found as white powders, as free diterpenes or even as acids or esters. Despite our result suggests the potential of polysorbate 80/sorbitan trioleate to form sucupira nanoemulsions, the surfactant to oil ratio (1:1) used in the present study probably was not sufficient to entrap and stabilize all these substances. Considering that surfactant to oil ratio is considered one of most important parameters that affect stability of nanoemulsions, especially for low energy methods [37], further studies may be performed to access its influence on sucupira oil based nanoemulsions formation and stability.

Preparation of nanoemulsion by low-energy methods, in contrast to high-energy methods, should be encouraged. These methods make use of chemical energy released due to a dilution process (self-emulsification methods) or make use of chemical energy released by phase transitions or change in surfactant curvature during the emulsification process, being able to induce formation of small droplets. Phase transitions can be induced by changing the temperature (PIT—phase inversion temperature method) or composition (PIC—phase inversion composition method) [20]. A great advantage of methods that involve low aport of energy is associated to reduction of process costs. We proved that sucupira nanoemulsions with good indicatives of physical stability could be obtained using this approach. Considering our aforementioned results, we opted to use the nanoemulsion prepared with polysorbate 80/sorbitan monooleate for biological investigation.

Table 2 shows mortality levels induced by sucupira nanoemulsion (expressed as sucupira oleoresin content). After 24 h, treatment with nanoemulsion at 25 mg/L was not considered statistically different from control group (p > 0.05). Group treated at 100 mg/L reached 26.67 ± 9.43% of mortality, which is significantly different from control group (p < 0.0001), treated groups at 25 mg/L (p < 0.01) and 200 mg/L (p < 0.0001). Higher mortality level was observed for group treated at 200 mg/L, which reached 86.67 ± 4.71% after 24 h and was considered significantly different from control and treated groups (p < 0.0001). After 48 h of treatment, statistically significant difference in mortality was observed for treated groups at 25 mg/L (p < 0.05), 100 mg/L (p < 0.0001) and 200 mg/L (p < 0.0001), when compared to control group. Lowest mortality level was observed for group treated at 25 mg/L (p < 0.0001), which reached 20 ± 0%. No statistically significant difference was observed in mortality levels induced by groups treated at 100 and 200 mg/L (p > 0.05), which reached 93.33 ± 4.71% and 100 ± 0%, respectively. Increased mortality levels were observed for groups treated at 25 mg/L (p < 0.1), 100 mg/L (p < 0.0001) and 200 mg/L (p < 0.01) as function of exposure time. *P. emarginatus* oil nanoemulsion presented median-lethal concentration (LC_50_) of 56.70 (30.12–94.97) mg/L after 48 h, in the larvicidal assay against *C. quinquefasciatus*. Other studies aimed to generate larvicidal herbal nanoemulsions against *C. quinquefasciatus*. The oil in water nanoemulsion prepared with neem oil decreased as function of droplet size as follows: 11.75 mg/L (mean droplet size around 31.03 nm), 25.99 mg/L (mean droplet size around 93.0 nm) and 62.89 mg/L (mean droplet size around 251.43 nm) [26]. After 24 h, it was observed that a mortality level below 40% was achieved with eucalyptus oil-based nanoemulsion at an experimental concentration of 50 mg/L [28]. Thus, our results are in accordance with mortality levels in this range of mean droplet diameter for classical larvicidal natural oils.

**Table 2:** Mortality levels of *Culex quinquefasciatus* after exposure to different concentrations of sucupira oil based nanoemulsion

Exposure time	Control	25 mg/L	100 mg/L	200 mg/L
24	0^a^	10 ± 0^a^	26.67 ± 9.43^b^	86.67 ± 4.71^c^
48	6.67 ± 4.71^a^	20 ± 0^b^	93.33 ± 4.71^c^	100 ± 0^c^

Data is expressed as mean ± standard deviation

Means in the same line with different superscript indicates statistical significant difference (P < 0.05)

Natural products have been recognized a valuable resource of potential larvicidal agents against disease vectors. Different criteria were proposed as standard for promising agents. Overall, satisfactory results are associated to samples that induce mortality levels higher than 75% [38] at 250 mg/L or have LC_50_ values below 100 mg/L [33], which are in accordance with our results. Optimized sucupira nanoemulsion, similar to the larvicidal nanoproduct that was used in the present study, was recently described as a promising larvicidal agent against *A. aegypti* larvae. It presented LC_50_ of 34.75 (7.31–51.86) mg/L [25]. Some plant extracts, including some obtained from species associated to diterpenoid-rich genus, revealed lower LC_50_ and LC_90_ values against *A. aegypti* larvae, when compared to *C. quinquefasciatus* larvae under same experimental conditions [39]. This data is in accordance with our results, which suggested that *C. quinquefasciatus* larvae are less susceptible to sucupira nanoemulsion than *A. aegypti* larvae.

**Fig. 2:**
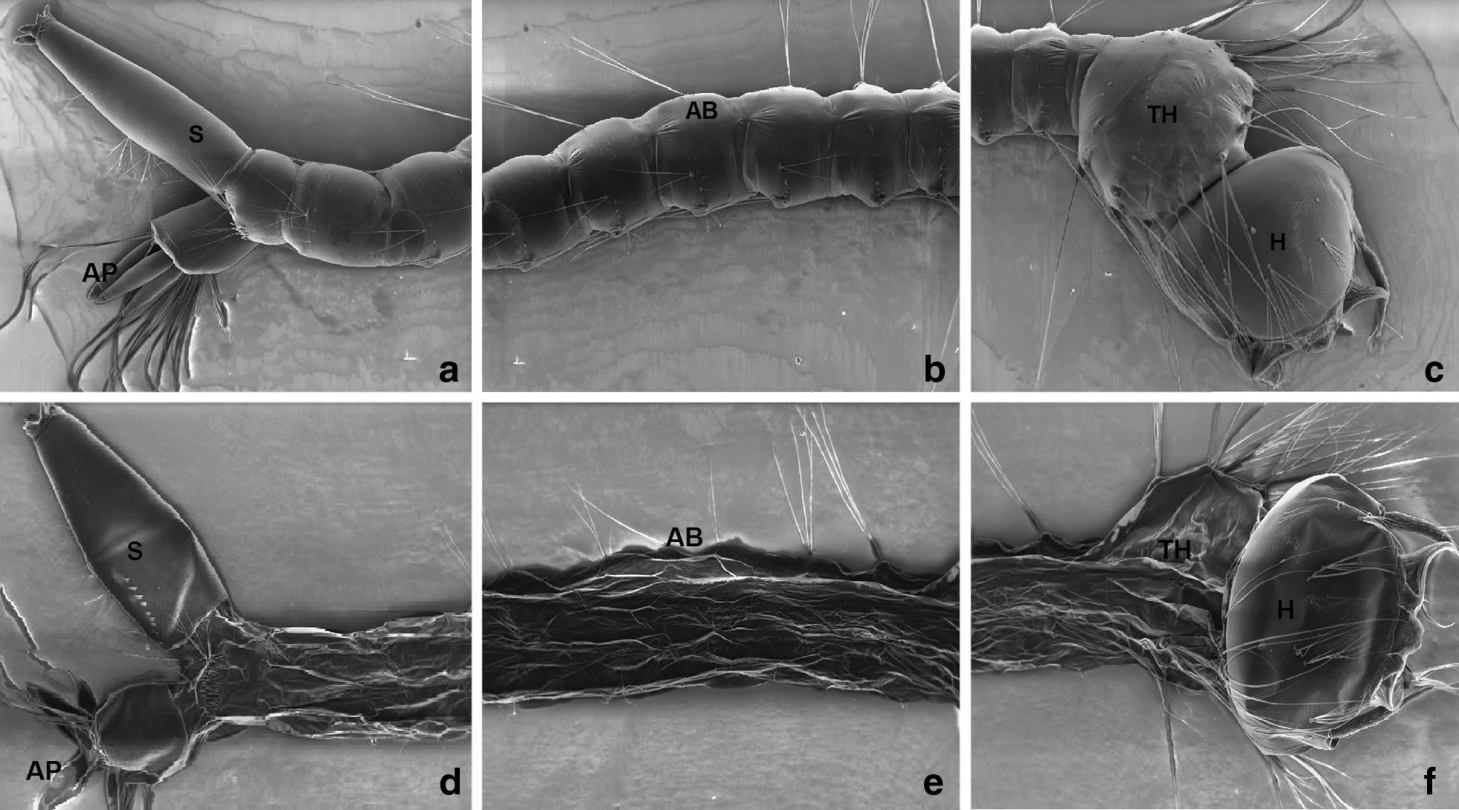
Scanning electron micrograph of C. quinquefasciatus larvae. Control (**a–c**) showing no alteration on head (H), thorax (TH), abdomen segments (AB), siphon (S) and anal papillae (AP). Larvae treated with P. emarginatusnanoemulsion at 250 ppm (**d–f**) showing alterations on cuticle of abdomen (AB), thorax (TH) and anal papillae (AP)

**Fig. 3:**
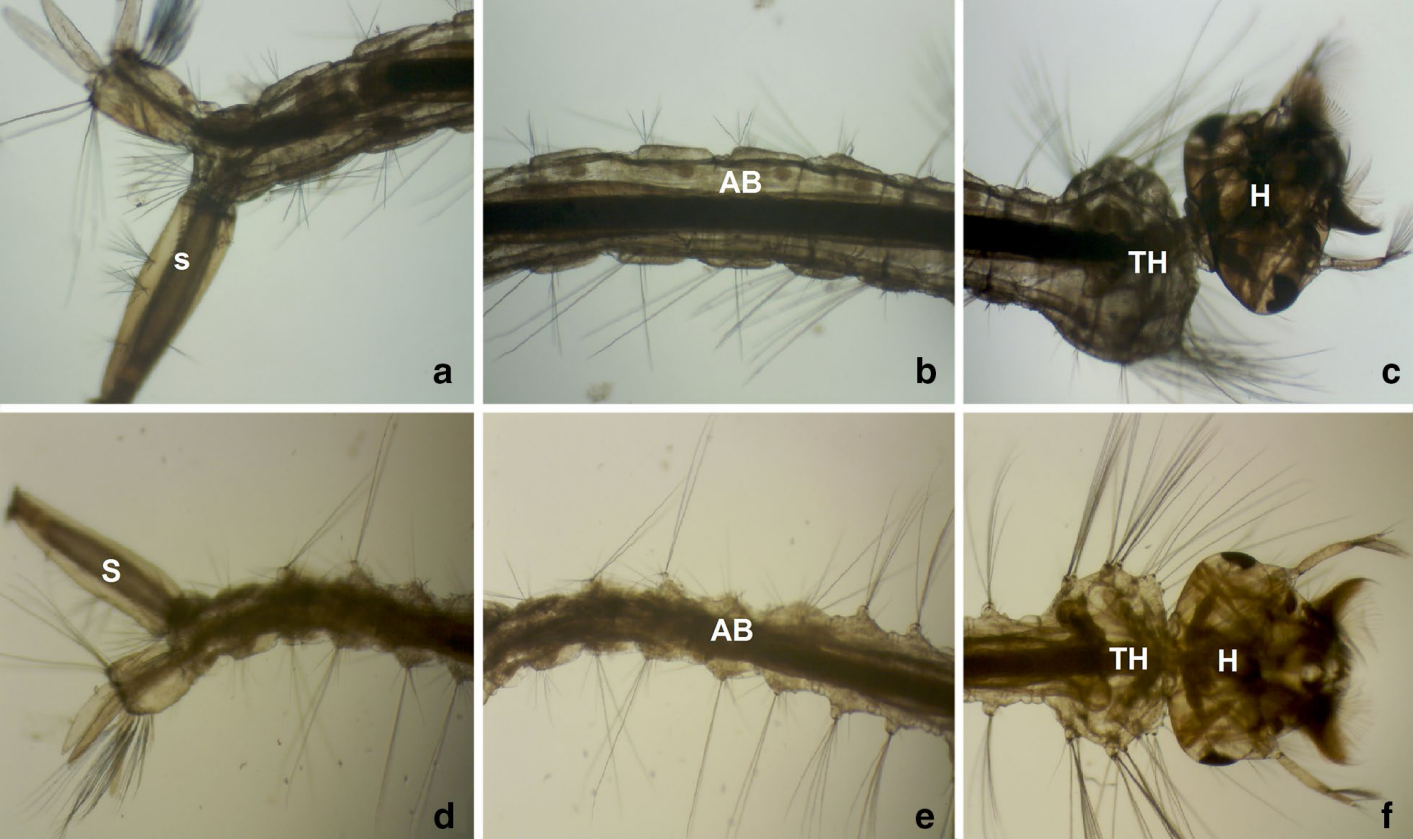
Light of C. quinquefasciatus larvae. Control (**a–c**) showing no alteration on head (H), thorax (TH), abdomen segments (AB), siphon (S) and anal papillae (AP). Larvae treated with P. emarginatus nanoemulsion at 250 ppm (**d–f**) showing alterations on the final abdomen segment (AB) (**e**)

Scanning electron micrography (Fig. 2a–c) and light micrography (Fig. 3a–c) shows that control larvae present normal appearance of following morphological regions: head (H), thorax (TH) and abdomen segments (AB). Moreover, no alterations of cuticle was observed on the control group. On the treated group, optical microscopy showed alteration only on the final abdomen segment (Fig. 3e). Regarding scanning electron micrography of the treated group, it was observed alteration on cuticle of abdomen region (AB), thorax (TH) and anal papillae (AP). Shrunken aspect and absence of perception of abdomen segments was observed (Fig. 2d–f). Similar observation on anal papillae of *Aedes aegypti* treated with extracts from some *Piper* species [40]. However, larvae from treated group did not exhibit major damage on siphon (S) and cephalic capsule (H). The observed alterations may affect larvae motility and my contribute partially to the observed activity. However, other factors are considered potentially responsible by larvicidal activity on mosquitoes, such as damage to digestive tube [41], which is associated to anti-feedant behavior [42].

**Fig. 4:**
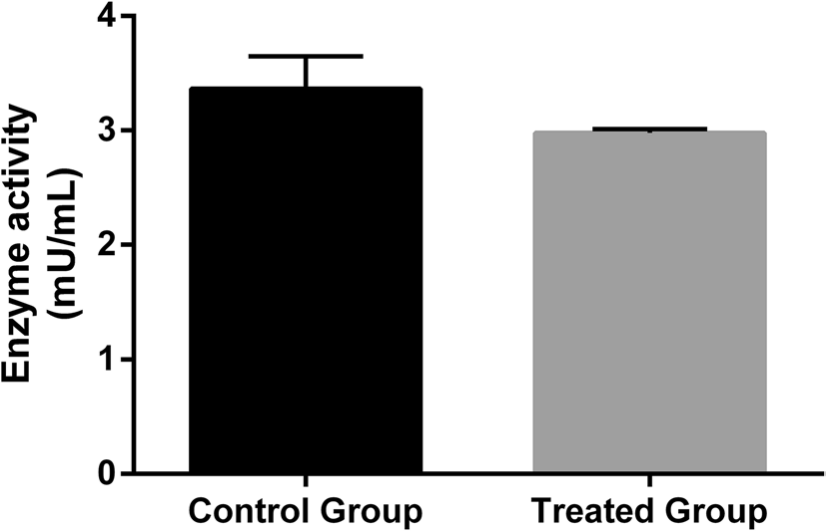
Acetylcholinesterase activity of enzyme from whole body homogenates of *Culex quinquefasciatus* larvae

**Fig. 5:**
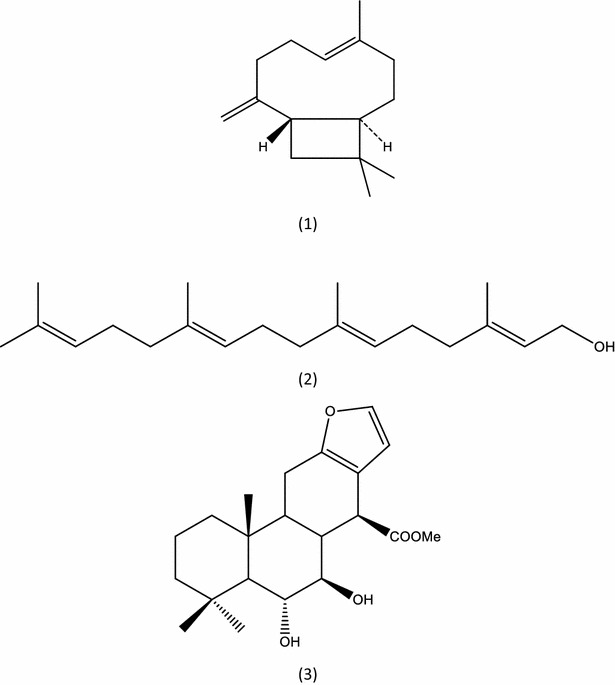
Chemical structures of the terpenoids found on *P. emarginatus* oleoresin. Sesquiterpene: β-caryophyllene (*1*). Diterpenes: geranylgeraniol (*2*) and methyl 6α,7β-dihydroxyvouacapan-17-β-oate (*3*)

Several mechanism of action have been proposed for insecticidal natural products, including inhibition of acetylcholinesterase [17], which has been considered determinant for mortality of mosquitoes larvae [43]. No statistically significant difference was observed between acetylcholinesterase activity in the presence and absence of *P. emarginatus* nanoemulsion (p > 0.05) (Fig. 4). A larvicidal nanoemulsion prepared with eucalyptus oil was able to reduce about 80% of acetylcholinesterase activity of the enzyme from *C. quinquefasciatus* and this inhibitory activity may be, at least partially, attributed to 1.8-cineole (eucalyptol) [28]. Sucupira oleoresin which was used on the present study was previously characterized by our group and present the sesquiterpene β-caryophyllene (three isoprene units) and diterpenes (four isoprene units), such as geranylgeraniol and methyl 6α,7β-dihydroxyvouacapan-17-β-oate as remarkable compounds [25]. Chemical structures of these terpenoids, properly identified by comparison to authentic standards on *P. emarginatus* oleoresin which was used on the present study, are shown on Fig. 5. Eucalyptol is a monoterpene that is well recognized as a potent insecticidal agent and acetylcholinesterase inhibitor. However, geraniol, which also have two isoprene units and play a main role in diterpenes formation [44], has weak inhibitory activity against acetylcholinesterase, despite it has strong insecticidal activity [45]. In addition to the fact that bioactive substances may present significant differences in acetylcholinesterase inhibitory activities, differences attributed to insect enzymes inhibitory sites may be attributed to absence of anticholinesterase activity found in the present study. This hypothesis should also be considered since a similar nanoemulsion prepared with this natural raw material was able to inhibit acetylcholinesterase from the *A. aegypti* larvae [25]. Further studies to investigate another possible mechanism of action, in addition to quantification of levels of secondary metabolites released from sucupira nanoemulsion during acetylcholinesterase assay should be carried out to support these findings.

Environmental toxicology assay was carried out using the green algae *Chlorella vulgaris* subjected to different sucupira nanoemulsion concentrations (expressed as sucupira oleoresin content) (Table 3). We observed formation of a precipitate and loss of typical green color of the algae dispersion just afterwards addition of nanoemulsion at 1000 mg/L, while no change in macroscopical appearance was observed for groups containing nanoemulsion at 500, 100 and 25 mg/L. After 1 day of treatment, 50% of reduction in cell density was observed for the group containing nanoemulsion at 500 mg/L, while no viable cell was observed on the group containing nanoemulsion at 1000 mg/L. Significantly decrease in cell viability was observed after additional period of 24 h (p < 0.0001), reaching 12% of viable cells after 2 days of treatment. No viable cell was found for the group treated with nanoemulsion at 500 mg/L after 3 days. No statistically significant difference was observed for the group treated with nanoemulsion after 3 days of treatment (p > 0.05). However, significantly decrease in cell density was observed after 7, 14, 21 and 28 days (p < 0.0001), reaching 80.0 ± 0.0%, 62.7 ± 3.4%, 47.7 ± 4.0% and 13.3 ± 18.9% of viable cells, respectively. During 14 days, no significant difference (p > 0.05) in cell density was for the group containing nanoemulsion at 25 mg/L. Low decrease in percentage of viable cells was observed after 21 and 28 days (p < 0.0001). Cell count in control group revealed 16% of increase in cell density from day 3 to day 7 (p < 0.001), which was kept constant until a total of 14 days of treatment (p > 0.05). This result was statistically different when compared to group treated with nanoemulsion at 25 mg/L in the same period (p < 0.0001). This fact is probably associated to a spontaneous growth that was suppressed by constituents of sucupira nanoemulsion. However, we can conclude that no significant difference was observed after the end of the experiment between control and group tested at 25 mg/L (p > 0.05). *C. vulgaris* is a green microalgae that develop a main role in the aquatic ecosystem, being in the first level of the trophic chain. Moreover, it has been considered a promising agent for bioremediation due to its ability to degrade oil [46] and other contaminants, such as nonylphenol [47]. This organism has been considered valuable as a bioindicator for ecotoxicological studies. It has short life cycle and is easily cultured in laboratory, being also sensitive to toxicants, among other advantages [48, 49]. Nano-size may enhance toxicological effects, when compared to bulk material. Thus, evaluation of ecotoxicological impact of nanostructures should be encouraged, including aquatical toxicology using *C. vulgaris* as biological indicator [50]. Complexes of carbon nanotubes-diuron increased toxicity of the herbicide against *C. vulgaris* [51]. Another study observed ecotoxicological effects of cellulose nanofibers in *C. vulgaris* and suggested impact of carbon nanotubes on this algae [49]. Aqueous extract of soil containing zinc oxide nanoparticles did not induce any toxicological effect on this aquatic organism [52]. To our knowledge, this is the first report of evaluation of ecotoxicological assay for a proposed larvicidal natural product-based nanoemulsion against *C. vulgaris*. Our previous data suggests that sucupira oleoresin-based nanoemulsion is potentially safe mammals, considering a non-target toxicological assay performed with mice. Thus, it presented potential application at domestic environment [25]. However, a major problem for utilization of pesticides is the possibility of they being leached by water and reach the environment, such rivers, estuaries and ocean [53]. It is worth mentioning that this situation involves dilution of the pesticide agent. Thus, in addition to biodegradable nature of natural products, concentration of nanoemulsion (expressed as sucupira oleoresin content) in the environment will probably be not toxic for green algae, considering our results using this non-target model.

**Table 3:** Percentage of viable cells of the green algae *Chlorella vulgaris* subjected to different sucupira nanoemulsion concentrations (expressed as sucupira oleoresin content)

Day	Concentration (mg/L)				
	25	100	500	1000	control
1	100 ± 0^a^	100 ± 0^a^	50 ± 0^a^	0 ± 0^a^	100 ± 0^a^
2	100 ± 0^a^	100 ± 0^a^	12 ± 0^b^	0 ± 0^a^	100 ± 0^a^
3	100 ± 0^a^	100 ± 0^a^	0 ± 0^c^	0 ± 0^a^	100 ± 0^a^
7	100 ± 0^a^	80 ± 0^b^	0 ± 0^c^	0 ± 0^a^	116 ± 0^b^
14	100 ± 0^a^	62.7 ± 3.4^c^	0 ± 0^c^	0 ± 0^a^	116 ± 0^b^
21	80 ± 0^b^	47.7 ± 4.0^d^	0 ± 0^c^	0 ± 0^a^	80 ± 0^c^
28	70 ± 0^c^	13.3 ± 18.9^e^	0 ± 0^c^	0 ± 0^a^	70 ± 0^d^

Results are expressed as mean ± stander deviation. Means in the same column with different superscripts are significantly different (p < 0.05)

## Conclusions

Novel nanobiotechnology larvicidal agents using natural products from plant origin are very promising for vector control. *Culex quinquefasciatus* is responsible for transmission of filariasis, a neglected tropical disease. Our results suggest that optimal nanoemulsions may be prepared with different surfactants using a low cost, organic solvent-free and low energy simple method. Moreover, this prototype proved to be effective against *C. quinquefasciatus* and probably has low toxic effects to environment. Thus, it can be concluded that sucupira oleoresin-nanoemulsion is potentially an ecofriendly novel nanoproduct that can be useful in integrated control programs of vector control.

## Methods

### Chemicals

Sorbitan trioleate, sorbitan monooleate, polyethyleneglycol 400 dioleate, polyethyleneglycol 600 dioleate, polyethyleneglycol 400 monooleate, polyethyleneglycol 600 monooleate, polysorbate 80 and polysorbate 20 were purchased from Praid Produtos Químicos Ltda (SP, Brazil). Acetylthiocholine iodide (ATCI) and 5,5-dithiobis-2-nitrobenzoic acid (DTNB) were purchased from Sigma-Aldrich (St Louis, MO). Distilled water was used for general procedures.

### Obtainment of *P. emarginatus* oleoresin

Fruits from *Pterodon emarginatus* Vogel (Fabaceae) were obtained from Central Market of Goiânia—GO (Brazil). Identification of plant material was performed by Dr. José Realino de Paula and a voucher specimen was deposited at the Herbarium of Goiás Federal University (GO, Brazil) under the register number 41714. Oleoresin from *P. emarginatus* fruits was obtained by cold pressing using a mini mechanical press (MPE-40 ECIRTEC), weighed and hermetically stored in amber glass flask and kept at −20 °C until utilization.

### Emulsification method

Emulsification method was performed using low energy method [37] with some modifications [25]. Emulsions were prepared with sucupira oleoresin and surfactant (s) to oil ratio was 1:1. Final concentration of sucupira oleoresin or surfactant (s) on the emulsions was 2500 mg/mL. Oily phase was constituted by *P. emarginatus* oleoresin and different pairs of surfactants at rHLB of *P. emarginatus* oil (rHLB = 11) (Table 4). Surfactants and oil were mixed using magnetic stirring (400 rpm) for 30 min under controlled temperature using a water bath (80 ± 5° C). Aqueous phase was added through oily phase under constant magnetic stirring rate (400 rpm) and temperature gradually decreased to room temperature in approximately 30 min. System was stirred for 1 h and after this period, an additional amount of water was added to restore final mass (50 g).

**Table 4:** Composition of oily phase of *P. emarginatus* nanoemulsions

Formulation	Surfactants	Concentration (mg/L)
1	T80/S80	1560/940
2	T20/S80	1360/1140
3	T80/TS	1740/760
4	T20/TS	1540/960
5	MP600/DP600	840/1660
6	MP600/DP400	1380/1120
7	MP400	2500
8	T20/DP600	380/2120
9	T20/DP400	760/1740
10	T80/DP600	500/2000
11	T80/DP400	1540/960

*P. emarginatus* oil concentration was 2500 mg/L. Surfactant mixture final concentration was 2500 mg/L (rHLB = 11). Final mass of each formulation was 50 g. T80 = polysorbate 80. S80 = sorbitan monooleate. T20 = polysorbate 20. TS = sorbitan trioleate. DP400 = polyethyleneglycol 400 dioleate. DP600 = polyethyleneglycol 600 dioleate. MP400 = polyethyleneglycol 400 monooleate. MP600 = polyethyleneglycol 600 monooleate

### Nanoemulsion characterization

Droplet size, polydispersity index and zeta potential of the nanoemulsions were determined using a Zetasizer ZS (Malvern, UK). Each sample was diluted with distilled water (1:20) for analysis. Measurements were performed in triplicate and results were expressed as the mean diameter ± standard deviation.

### Larvicidal assay

*Culex quinquefasciatus* female were collected at Macapá (Universidade Federal do Amapá, Brazil), identified in the Laboratory of Arthropoda of Amapá Federal University and its eggs were used for the reared colony. Biological assay was performed under controlled conditions, being fourth-instar larvae kept at 25 ± 2 °C, relative humidity of 75 ± 5% and a 12 h light: dark cycle. Experimental protocol was performed according to WHO protocol [54] with some modifications. All experiments were performed in triplicate with 10 forth-instar larvae in each sample. Nanoemulsion was diluted in distilled water at 200, 100, 25 mg/L (expressed as sucupira oleoresin content on aqueous media). Control group was constituted by deionized water. Mortality levels were recorded after 24 and 48 h of exposure. If mortality level of the control was between 5 and 20%, correction of mortality levels of treated groups should was performed using Abbott´s formula as follows: Mortality (%) = 100 (X−Y)/X, where X = percentage survival in the untreated control and Y = percentage survival in treated sample.

### Morphological study

After treatment, larvae was fixed on ethanol 70% and analyzed under light microscopy (Mod. BX41, Olympus,) and photographed with a camera MDCE 5C. External morphology was also evaluated under low vacuum using a Tabletop Microscope TM3030Plus (Hitachi, Japan).

### Enzymatic assays

Whole body homogenate was prepared according to previously established method [28]. Larvae from control group was collected and water was gently removed using tissue paper. Then, they were separately homogenized with 3.0 mL phosphate buffered saline (PBS) 0.1 M (pH = 7.5). This step was performed using a T25 Ultra-Turrax homogenizer (Ika-Werke, Staufen, Germany) running at 12,000 rpm for 1 min. The homogenate was centrifuged for 30 min (5000 rpm) under controlled temperature (10 °C). Whole body homogenate supernatants were collected and immediately used for enzymatic assay.

Anticholinesterase activity was performed according to the well-established method described by Ellman et al. (1961) [55] with some modifications. Activity of acetylcholinesterase from whole body homogenate, after exposure to optimized *P. emarginatus* nanoemulsion, was determined as follows: Aliquot of 0.25 mL of this nanoemulsion, 0.25 mL of whole body homogenate supernatant and 0.5 mL of DTNB were added to 1.75 mL of phosphate buffer. The mixture was incubated for 10 min (25 ± 1 °C). Then 0.25 mL of ATCI was added and the absorbance was measured at 410 nm using a UV-Mini spectrophotometer (Shimadzu). Maximum acetylcholinesterase activity was achieved by replacing the amount of *P. emarginatus* nanoemulsion by PBS. Blank was obtained by replacing the ATCI by a same amount of PBS. Assays were performed in triplicate and results were considered significant when (p < 0.05).

### Environmental toxicology assay

The green algae *Chlorella vulgaris* was isolated from water samples obtained from Lagoa dos Índios, situated on the municipality of Macapá (latitude 0.031368 and longitude 51.102559). Serial dilution was carried out in order to isolate the colony and cells were inoculated into NPK media. Algae counting was carried out using a Neubauer chamber [56]. This organism was used as a non-target model for environmental toxicology assay. Aliquot of 10 ml of *C. vulgaris* inoculum was cultivated in nitrogen/phosphorus/potassium (NPK, 08:08:08) aqueous solution. Initial cell density was 1 × 10^6^ cell/mL for all tested groups. Nanoemulsion was tested at different concentrations (25, 100, 500 and 1000 mg/L, expressed as oleoresin content). Control group was constituted by *C. vulgaris* dispersion (1 × 10^6^ cel/mL) and NPK aqueous solution. Cell count was performed after 1, 2, 3, 7, 14, 21 and 28 days. Percentage of viable cells (%VC) was calculated as follows: %VC = (D/D_0_) × 100, where: D is cell density before nanoemulsion addition, D_0_ is cell density after at each specific day.

### Statistical analysis

Analysis of variance (Two-way ANOVA) followed by Tukey´s test or Bonferroni´s test was conducted using the Software GraphPad Prism 6.0 (San Diego, California, USA). Differences were considered significant when p ≤ 0.05. Probit analysis was performed with 95% confidence interval for LC_50_ determination.
